# Enhanced postoperative cancer therapy by iron-based hydrogels

**DOI:** 10.1186/s40824-022-00268-4

**Published:** 2022-05-23

**Authors:** Haomeng Zhang, Meng Zhang, Xinyu Zhang, Yuan Gao, Yanling Ma, Hongyu Chen, Jipeng Wan, Changzhong Li, Fei Wang, Xiao Sun

**Affiliations:** 1 Department of Gynecology, Shandong Provincial Hospital, Shandong University, 250021 Jinan, China; 2 School of Chemistry and Pharmaceutical Engineering, Medical Science and Technology Innovation Center, School of Radiology, Shandong First Medical University & Shandong Academy of Medical Sciences, 250000 Jinan, China; 3 Department of Chemical and Biomolecular Engineering, National University of Singapore, 117585 Singapore, Singapore; 4 Department of Gynecology, Shandong Provincial Hospital Affiliated to Shandong First Medical University, 250021 Jinan, China

**Keywords:** Iron-based hydrogels, Biocompatibility, Synergistic cancer therapy, Postoperative

## Abstract

Surgical resection is a widely used method for the treatment of solid tumor cancers. However, the inhibition of tumor recurrence and metastasis are the main challenges of postoperative tumor therapy. Traditional intravenous or oral administration have poor chemotherapeutics bioavailability and undesirable systemic toxicity. Polymeric hydrogels with a three-dimensional network structure enable on-site delivery and controlled release of therapeutic drugs with reduced systemic toxicity and have been widely developed for postoperative adjuvant tumor therapy. Among them, because of the simple synthesis, good biocompatibility, biodegradability, injectability, and multifunctionality, iron-based hydrogels have received extensive attention. This review has summarized the general synthesis methods and construction principles of iron-based hydrogels, highlighted the latest progress of iron-based hydrogels in postoperative tumor therapy, including chemotherapy, photothermal therapy, photodynamic therapy, chemo-dynamic therapy, and magnetothermal-chemical combined therapy, etc. In addition, the challenges towards clinical application of iron-based hydrogels have also been discussed. This review is expected to show researchers broad perspectives of novel postoperative tumor therapy strategy and provide new ideas in the design and application of novel iron-based hydrogels to advance this sub field in cancer nanomedicine.

## Introduction

Cancer is one of the diseases with the highest mortality rate to severely obstruct life expectancy expansion and life quality improvement [1, 2]. According to the statistics of the World Health Organization in 2019, the number of patients who died from cancers have exceeded cardiovascular and cerebrovascular accidents, in more than 60% countries, to become the first or second leading cause of death of the group younger than 70 [3]. Surgery is the commonly used method to treat solid tumors [4]. However, the surgical resection cannot eradicate carcinoma tissue completely which poses a challenge to the clinical treatment. Some cancer focuses may inevitably remain on the wound tissue because of the infiltrative and invasive properties may significantly increase the risk of local recurrence and metastasis [5–8]. Some researchers considered that surgery enhances successful implantation of spilled tumor cells [9–11]. Besides, in clinical practice, eliminating cancerous tissue inevitably leads to large tissue defects, which not only impairs normal function and appearance, but also causes impact to the mental health of patients [12–16]. Meanwhile, the surgery will cause potential bacterial infection to impede wound healing and skin regeneration in the resection site [17–21]. Therefore, it is urgently needed to develop new therapeutic methods to meet the requirements of comprehensive treatment after radical surgery of tumor.

To eradicate residual tumor cells and reduce recurrence rates, radiotherapy and chemotherapy are generally used as adjuvant treatments alongside with surgery [22]. Systemic application of chemotherapy drugs is preferred with relatively higher effectiveness, especially in the treatment of hardly approachable or unknown tumor, because the drugs can affect cancer cells even outside their primary region [23]. However, conventional systematic chemotherapy still faces some challenges, mainly including low efficacy to reach the tumor sites and the ensuing adverse reactions [24–26]. Consequently, local chemotherapy would be beneficial, particularly in the case of approachable tumor tissue. In the drug-loaded system, drugs can be directly located at the cancerous region to avoid excessive drug circulation compared to systemic drug administration with suppressed serious side effects on normal tissues [27–32]. In local drug delivery systems, hydrogel-based cancer therapy platforms have attracted much attention. Hydrogel is a network polymer formed by crosslinking gel monomers with soft texture, excellent biocompatibility, adjustable biochemical and biophysical properties [33–36]. There are many kinds of hydrogels with different synthesis methods to meet a variety of application requirements [37]. Here, the advantages of hydrogel in postoperative treatment of tumors are listed. Firstly, high drug loading capacity [38–40] and good compatibility in drug co-encapsulation [41–47] can endow the system different functions and therapeutic effects. Drug-loaded hydrogels can be filled into the tumor site or cavity after the surgical resection of solid tumor to maintain the local drug concentration for a long time and greatly alleviate the possibility of recurrence of residual cancer cells [48]. Secondly, hydrogel can realize controllable drug releasing [49–51]. Hydrogels can respond to various stimuli [52], for example, light [53], near infrared light (NIR) [54–56], temperature [57, 58], pH [59], magnetic field [60], enzymes [61], ionic strength and electric field. The controlled release of drugs can be realized by virtue of the reversible expansion and contraction properties of hydrogels under specific stimulation conditions to fix the size of gel grids. This customizable antineoplastic protocol makes it possible to achieve more personalized cancer therapy. Thirdly, hydrogels also have great clinical practice potential in tissue reconstruction. As a powerful biomaterial, hydrogel has been widely used in tissue engineering, including spinal cord repairment [62], wound healing [63], tissue reconstruction [64, 65], and bone regeneration [66]. For example, hydrogel has unique advantages in the process of tissue reconstruction and plasticity after breast-conserving surgery for breast cancer [67]. After the visible lesions are surgically removed, hydrogels can be used to fill and repair the breast defects to inhibit tumor recurrence while restoring its original appearance. On the other hand, the hydrogel has good water absorption, moisture retention characteristics and air permeability to provide biomechanically supportive 3D microenvironments for cell proliferation and migration, showing potential application prospect in the field of serving as external auxiliary materials [68]. These dominant positions boost the development of hydrogel for comprehensive tumor therapy after surgery.

Polymer hydrogels can be prepared in different methods that can be classified into chemical or physical hydrogels depending on the cross-linking mechanism. The chemical or permanent hydrogels are formed by covalent crosslinking of polymer chains. While physical crosslinking hydrogel are made via hydrogen bonding, crystallization, hydrophobic interaction, entangled chains, ionic interaction, and coordination bond, giving them a reversible character [69]. In recent years, reasonably designed metal coordination cross-linked hydrogels by introducing metal ions have aroused wide interest among researchers. The doped inorganic metal ions, such as Cu [24, 70], Ag [71], Au [66], Zn [72], Fe and other metal elements, can endow hydrogels abundant biological functions [73]. Among them, Fe is an essential nutrient element for human body [74] and possesses good biocompatibility, playing a vital role in a multitude of biochemical reactions [75–82]. The addition of Fe element in hydrogel has been shown diversified curative effects, enabling it to meet various demands. In the experiment of synthesizing artificial muscle with hydrogel, Fe ions and other forming structures can enhance the strength of hydrogel, give it a certain contractility [83] and achieve self-recovery function [84]. Besides, Fe can be reduced to Fe^2+^ or Fe^3+^ in tumor or bacterial microenvironment to trigger Fenton reaction, and the produced hydroxyl radical (•OH) can effectively kill cancer cells and avoid infection in postoperative adjuvant therapy [85, 86]. Fe also has photothermal property. By in-situ heating, the hydrogel shows a significant promoting effect on angiogenesis and chronic wound healing in vivo [87, 88]. Meanwhile, Fe^2+^ itself shows the function in enhancing the proliferation of HUVEC and up-regulating the expression of endothelial cell-related genes such as vascular endothelial growth factor (VEGF) and Endothelial Nitric Oxide Synthases (eNOS) [89]. Fe^3+^ can contribute to the enhancement of the cohesion and interface adhesion that can be used in myocardial infarction recovery [90]. In addition, Fe makes great progress in tissue engineering, like assisting the stem cells proliferation and differentiation [91], promoting liver [92] and bone [93] regeneration, constructing artificial livers [92] and so on. Iron-based hydrogel can also be used for topical treatment, such as wound disinfection [94] and tendon tissue injury recovery [95]. What’s more, Fe has a great potential in adjuvant treatment after surgery for malignant tumors [96–102]. For example, with the help of the para-magnetism of Fe ions, under the external magnetic field, heat can be released to achieve the directional killing of tumor cells [103].

In general, hydrogel is a new type of biomaterial with great clinical application and development potential which can provide individualized synthesis in the fields of residual lesion removal, anti-infection, promotion of tissue healing, and reconstruction of defective tissue after tumor surgery. However, hydrogels still have many technical and conceptual challenges are waiting to be solved before successful clinical applications. For example, its biosafety, biodegradability, metabolic rate, and cytotoxicity require further study. At this stage, the short-term biocompatibility of hydrogels is only evaluated in animal models without human clinical trials to verify its effectiveness and long-term safety. Secondly, the common use of hydrogels is topical application. Compared with the mature treatment methods such as intravenous injection and oral administration, hydrogels require operators with higher technical operant level which may cause a negative impact on the promotion of hydrogels. Furthermore, hydrogels are composed of multiple component that can be applied on multiple therapeutic strategies, but the complicated system design obstacle its scale-up and quality control.

Overall, in this review, the latest progress of iron-based polymeric hydrogels with a three-dimensional (3D) network structure in postoperative tumor therapy have been highlighted, including chemotherapy, photothermal therapy (PTT), magnetic hyperthermia therapy (MHT), photodynamic therapy (PDT), chemo-dynamic therapy (CDT), and multimodal combination therapy. In addition, the advantages and limitations of these iron-based hydrogels in the categorized examples have been discussed and emphasized. Finally, the future directions of novel strategies to fabricate iron-based hydrogels will be reconsidered in the aspect of biosafety which are the key concerns related to medical translation. This review could hopefully offer researchers a broader perspective of iron-based hydrogels for the enhanced postoperative tumor therapy and provide new ideas in the design and application of novel iron-based hydrogels.

### Application of Iron-based hydrogels for postoperative cancer therapy

**Fig. 1:**
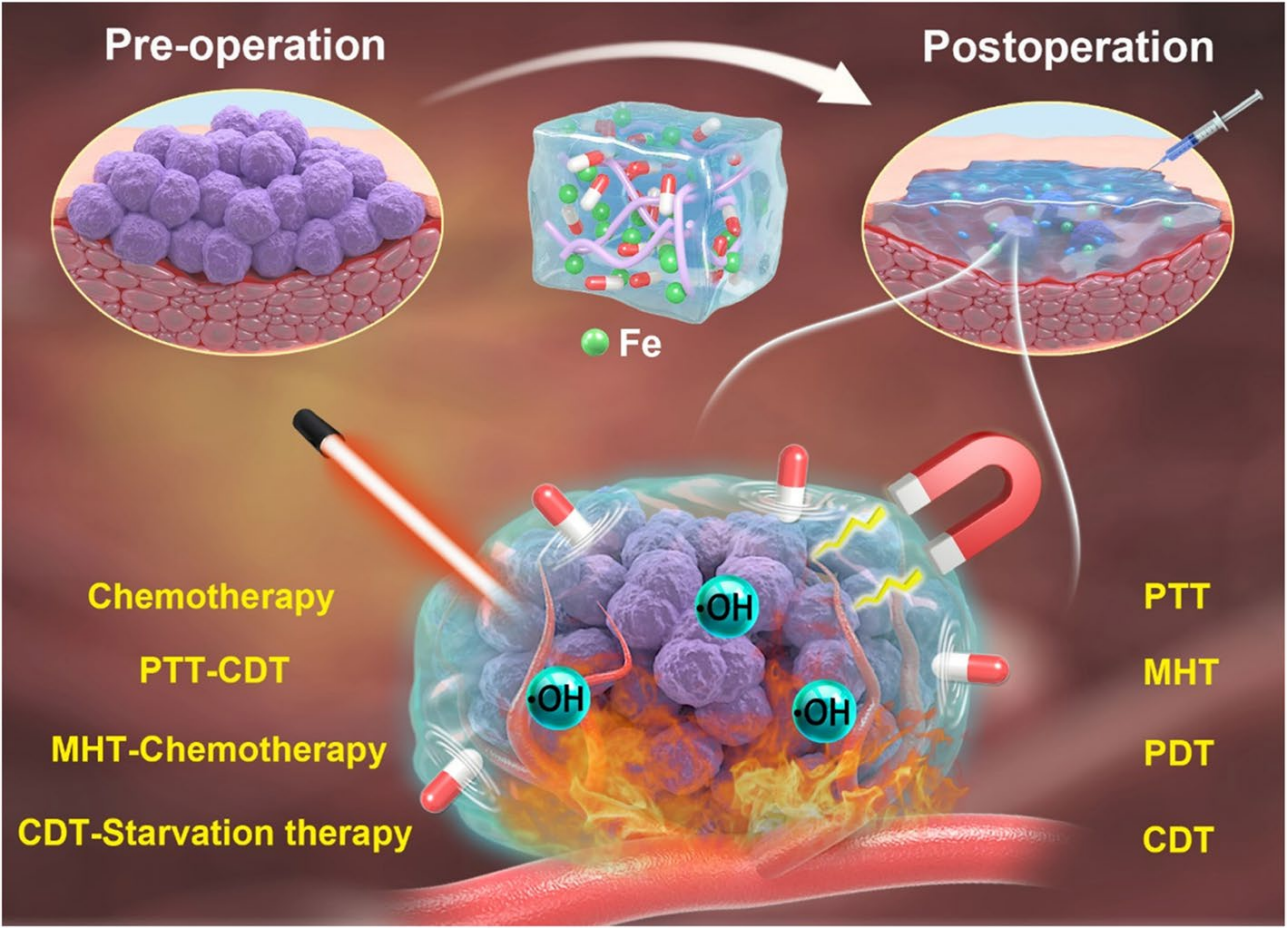
Iron-based hydrogels for postoperative cancer therapy

Chemotherapy have been widely used in postoperative tumor therapy, while the introduction of hydrogel platform greatly improves the efficacy of chemotherapy and potentially reduces systemic toxicity. In the last decades, with the rapid development of nanomedicine, new cancer treatments such as PTT, MHT, PDT, and CDT have attracted extensive attention due to their unique advantages including high specificity, low aggressiveness, and precise spatiotemporal selectivity. Simultaneously, the current trend in clinical studies has gradually shifted from a single treatment modality to the combined or multiple therapies since the synergistically enhanced interactions between two or more treatments results in significant super additive (namely “1 + 1 > 2”) therapeutic effects. Notably, due to the inherent advantages of Fe ions, many iron-based hydrogel platforms with integrated multiple treatments and functions have been established (Fig. 1) which has shown great application prospects in postoperative tumor therapy. Here in Table 1, some research publications in the recent years of iron-based hydrogel applied in postoperative treatment are listed.

**Table 1:** Research publication in the recent years of hydrogel for cancer treatment

Host material	Existence	Function	Indication	Treatment strategy	Ref.
Chitosan-catechol based hydroge	Fe^3+^	realizing cohesive interaction and sequential release of drug	Lung and breast cancer	Chemotherapy	[96]
Magnetic supramolecular hydrogel	Fe_3_O_4_	thermally inducing cell damage; realizing triggered releasing of chemo- therapeutic drugs	breast cancer	MHT-Chemotherapy	[97]
Citrate-iron hydrogel scaffold	Fe^3+^	photothermal response; decreasing the inflammation response; improving angiogenesis; tissue regeneration	skin cancer	PTT	[98]
PEGDA and AIPH	BGN-Fe-Ag_2_S	photothermal effect and chemodynamic effect; eliminating multidrug resistant bacteria; accelerating wound healing	breast cancer	PTT-CDT	[99]
Chitosan-based dynamic hydrogels	FVIOs	generating heat and maintaining rheological integrity; promote DOX to enter the nuclei of cancer cells	breast cancer	MHT-Chemotherapy	[100]
DOX-loaded magnetic alginate-chitosan microspheres	SPIONs	realizing magnetic hyperthermia agents and drug release triggers	breast cancer	MHT-Chemotherapy	[101]
Gallic acid-ferrous	Fe^3+^	realizing NIR absorbing Fenton catalyst	breast cancer	CDT-starvation therapy	[86]
Hyaluronic acid-gallic acid	Fe^3+^	absorbing NIR light energy to vibrating heat energy	skin and breast cancers	PTT	[104]

#### Iron-based hydrogel for chemotherapy

**Fig. 2:**
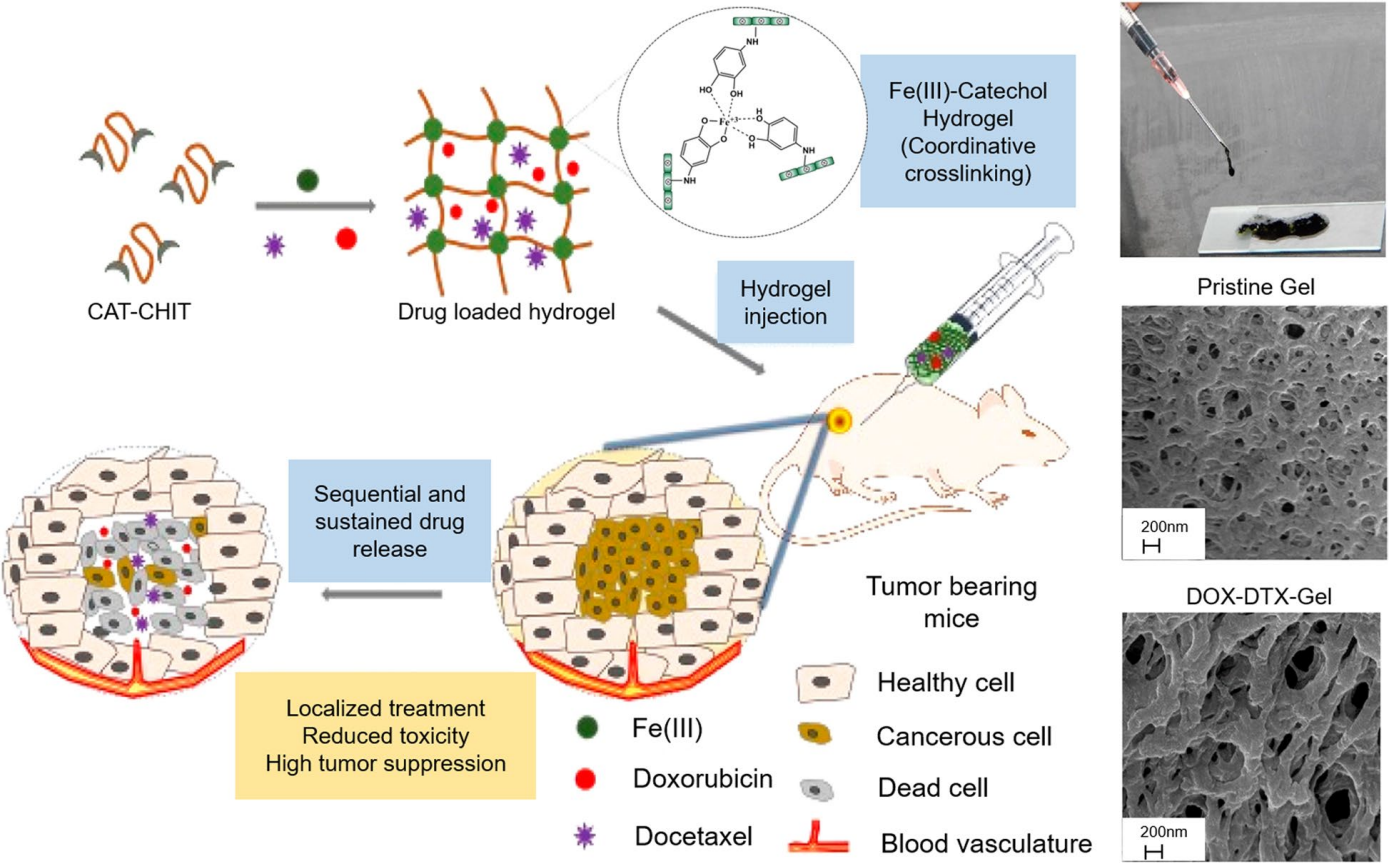
Schematic illustrations of the synthesis of Fe (III)-catechol hydrogels and their potential application in inhibiting tumor recurrence by sequential release of chemotherapeutic drugs. Reproduced with permission from Ref. [96]

Fe can promote the hydrogel aggregation in different ways to enhance the cohesion of the hydrogel and induce chelating to achieve the controlled release of drugs at the specific target. Recently, iron-based hydrogel as a novel drug delivery system have been developed to deliver a wide range of chemotherapeutic drugs. Yavvari et al. synthesized chitosan-catechol based hydrogel (CAT-CHIT) that was assembled by catechol-Fe (III) coordination interaction to achieve the effective delivery of chemo-therapy drugs (Fig. 2). CAT-CHIT contains two kinds of catechol which form different complexes with Fe (III) in different structures respectively. When the polymer solution is at an optimal Fe(III)-catechol molar ratio of 1:3, the cohesive interaction is necessary for hydrogel to be detected. At the same time, Fe (III) has a strong chelating ability with doxorubicin (DOX), and the formation of CAT-CHIT polymer become faster with the addition of Fe (III). In the drug release experiments, DOX was firstly released to maintain a high concentration of drug around the tumor, however the release was very slow due to the strong interaction of docetaxel (DTX) with hydrogel network which achieved the sequential release of drug [96]. The hydrogel has an ideal function of realizing comprehensive post operation treatment through chemotherapy, the competitive coordination ability of Fe(III) ions can target the goal of sequential, continuous and local delivery of two different chemotherapy drugs in the gel implanted near the tumor, and the self-healing property of the hydrogel can prolong the half-life of drug metabolism and remarkably improve the median survival rate of affected mice.

**Fig. 3:**
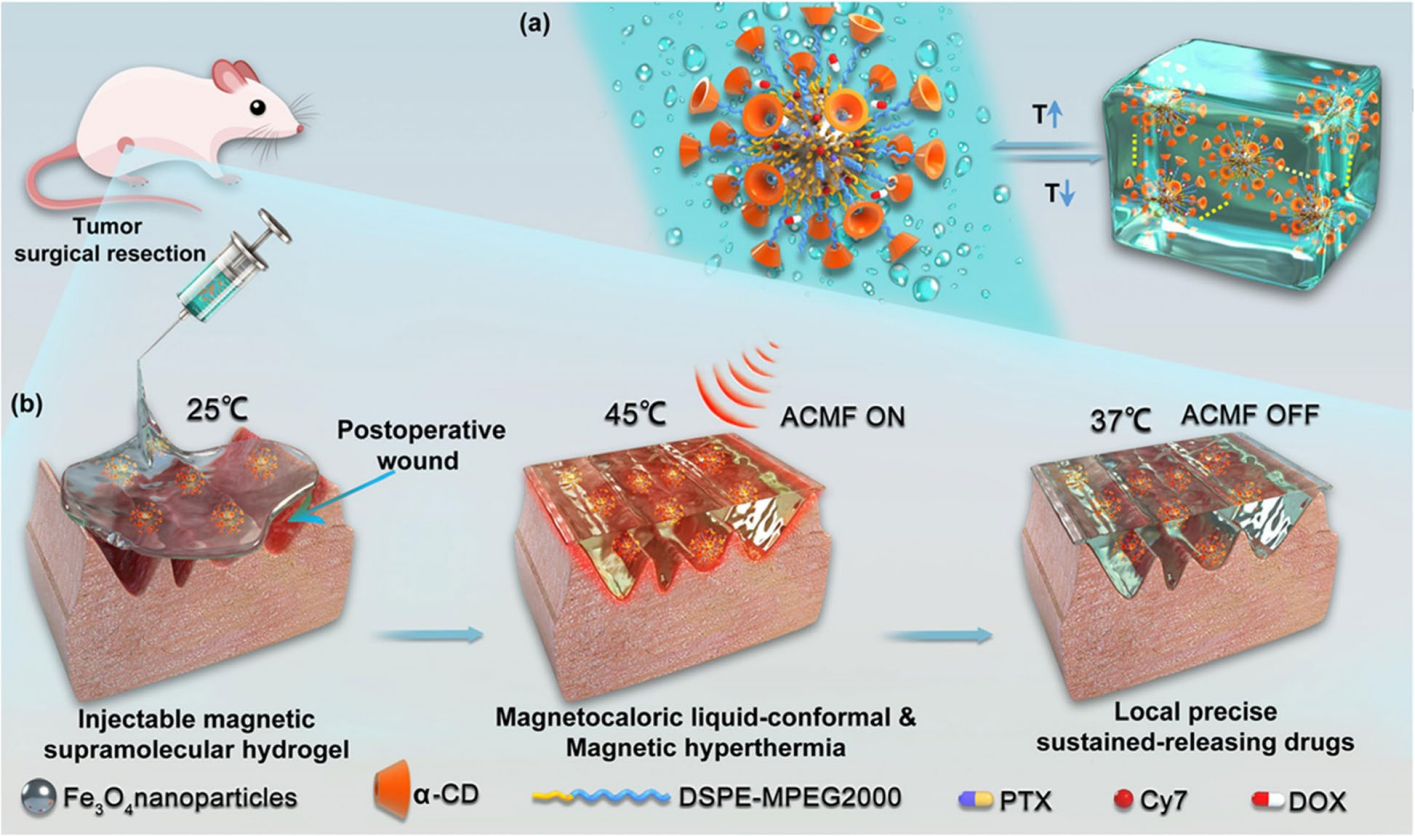
Schematic illustrations of the synthesis process of MSH and their application of the MSH in inhibiting tumor recurrence and wounding healing under the ACMF. Reproduced with permission from Ref. [97]

In addition, as a magnetic species, under the premise of giving an external magnetic field, Fe can generate heat and enhance the drug release. Wu et al. designed a new type of magnetic supramolecular hydrogel (MSH) that was self-assembled by PEGylated Fe_3_O_4_ nanoparticles and α-CD through the inclusion complexation. It was capable for inhibiting the locoregional recurrence of cancer following the primary tumor resection in a breast mouse model (Fig. 3) [97]. In the layered structure, with Fe_3_O_4_ as the core, this structure ensures that drugs of different properties can be continuously delivered with different release profiles, so that hydrophobic molecule paclitaxel (PTX) and hydrophilic molecule DOX can be loaded simultaneously. MSH is gel-like under 37 ℃, after being injected into the body, the heat released from Fe_3_O_4_ by alternating current magnetic field (ACMF) can transform MSH from gel to liquid to match the irregular cavity after tumor resection and exert the therapeutic effect on tumor cells to the maximum extent by covering the whole residual tumor. Thermal generated under the ACMF by Fe_3_O_4_ promotes the release of DOX and PTX, significantly increases the cumulative release and maintains the effective therapeutic concentration ultimately. Besides, induction thermal mediated by magnetic iron nanoparticles provides the effect of thermal-induced cell damage. MSH has many advantages, for example, MSH with the characteristic of shear thinning can be easily injected into a surgical site through a needle. After being exposed to ACMF, hydrogel can perfectly fill the tissue cavity after tumor surgery through magnetothermal gel-sol transformation. The magnetic induced thermal effect promotes the controlled release of chemotherapy drugs at different rates, so that recurrence and metastasis of tumors are prevented in various methods.

#### Iron-based hydrogel for PTT

**Fig. 4:**
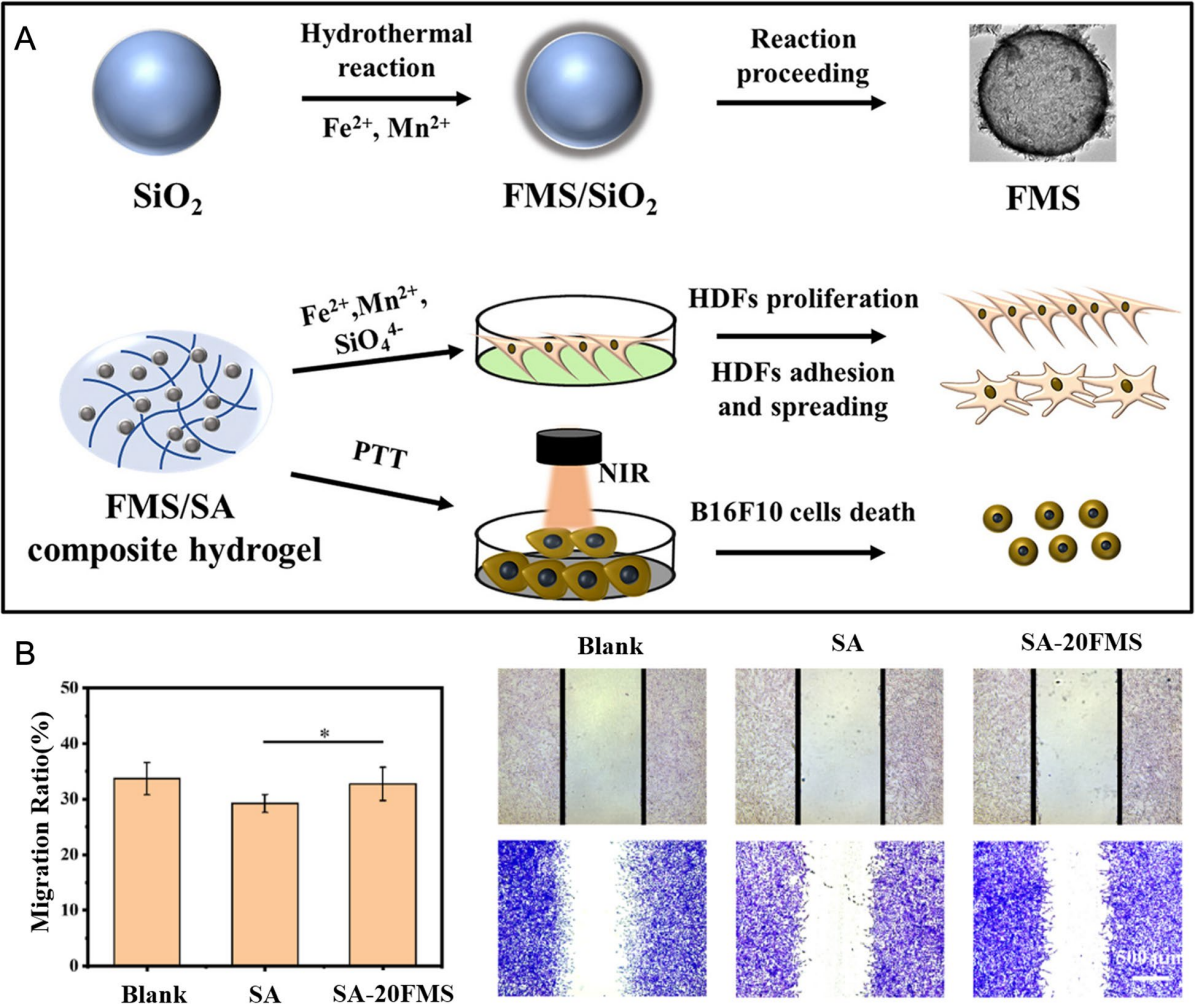
Schematic diagram of action mechanism and curative effect of FeMn(SiO_4_)-incorporated bioactive hydrogels. **A** Schematic illustrations of the preparation of FeMn(SiO_4_)-incorporated bioactive hydrogels and their application in postoperative cancer therapy. **B** The migration performance of human dermal fibroblasts (HDFs) on various time (0 h, 12 h) and the corresponding migration rate analysis (*p < 0.05). Reproduced with permission from Ref. [110]

PTT has been considered as an effective method for antitumor and anti-infection due to its high efficiency, high selectivity, and low side effects [105–109]. Since the rapid proliferated tumor cells are more sensitive to temperature raise than normal cells, increasing temperature within a certain range could kill tumor cells without affecting others. In the experiment of Ma et al., an iron manganese silicate (FeMn (SiO_4_), FMS)-incorporated bioactive hydrogel was synthesized [110]. The synthesized FMS owns a hollow spherical structure with a rough surface and exhibits a good photothermal effect which is presumably attributed to its own olivine structure (Fig. 4A). Under the irradiation of NIR, FMS can absorb the photon energy and interact with the lattice vibration to enhance the lattice thermal vibration, and finally generate thermal through phonon scattering. Half of the iron atoms in Fe_2_SiO_4_ are occupied by Mn^2+^ which will lead to lattice distortion to a certain extent, thus enhancing the thermal vibration of the lattice under laser irradiation and improving the photothermal performance. In addition, the Fe released by FMS can promote the expression of VEGF, accelerate cell proliferation and migration (Fig. 4B), and promote wound healing. Therefore, FMS/SA composite hydrogel has good biological activity and can be used as a biological material for skin regeneration. Ma et al. believed that no one had studied FMS in the photothermal therapy field before their experiment. After FMS was mixed into sodium alginate hydrogel, a hydrogel with potential application value in the treatment of tumor-induced skin wound defect was obtained. FMS/SA composite hydrogel can play an ideal role in the comprehensive treatment of melanoma after resection with a good development prospect. However, there are still some limitations in this experiment. For example, the photothermal effect of FMS/SA composite hydrogel and its biological activity of promoting wound healing can only be verified by in vitro experiments without animal experiment to show the simulation of this hydrogel in animal or human body.

**Fig. 5:**
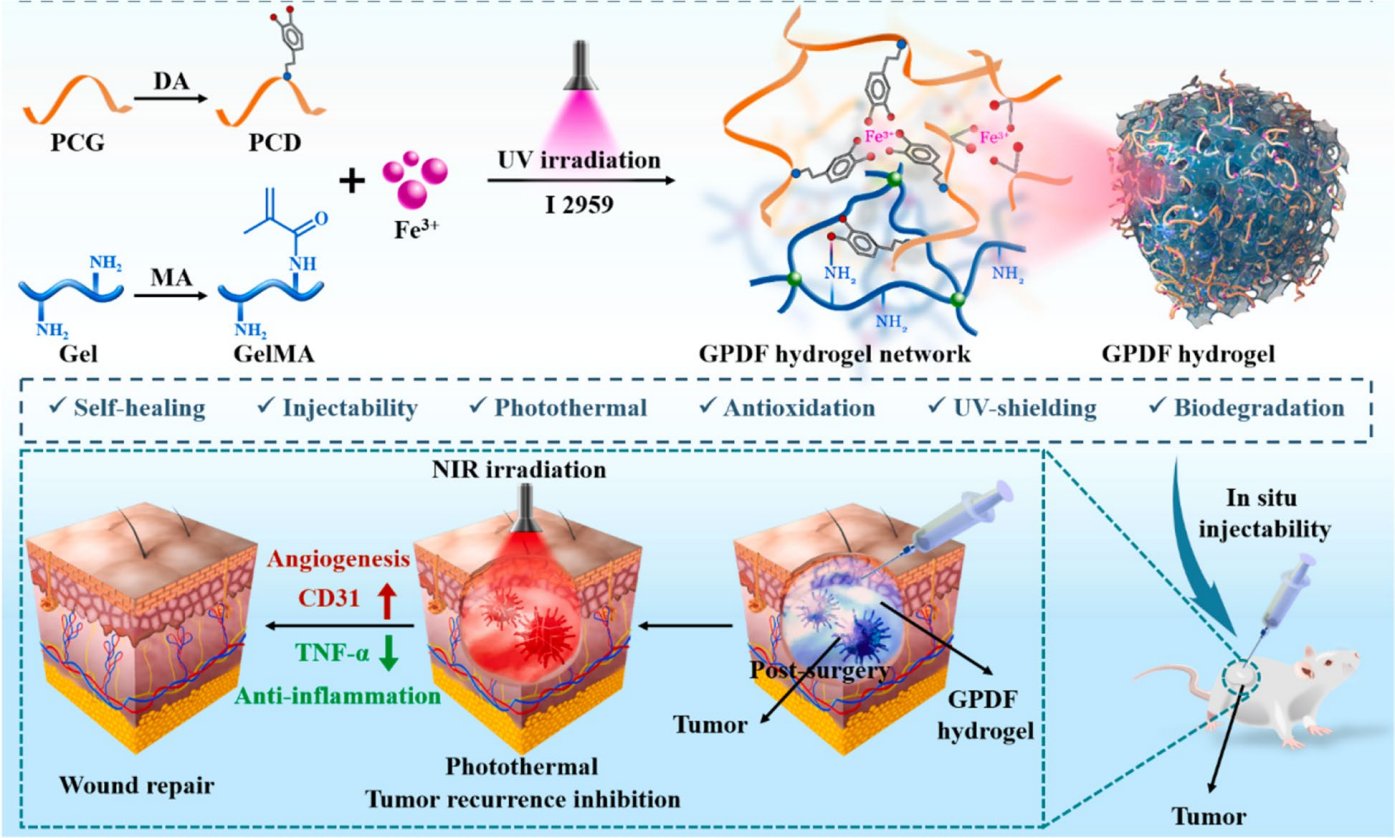
Schematic illustration for the fabrication process of GPDF hydrogel and the potential application of inhibiting tumor recurrence and promoting wound healing. Reproduced with permission from Ref. [98]

Luo et al. synthesized a multifunctional bioactive therapeutics-repair-enabled citrate-iron hydrogel scaffold (GPDF) used for comprehensive post operation treatment of skin cancer (Fig. 5) [98]. In the presence of poly (citric acid-ethylene glycol) (PCG)-dopamine (PCD) and Fe^3+^, gelatin/methacrylic anhydride (GelMA) was photo-crosslinked to prepare a double-network GPDF hydrogel. Attributed to the double-network physical cross-linking of catchol-Fe^3+^ coordination and hydrogen bond between GelMA and PCD polymers, GPDF hydrogel obtained the ideal injectable and self-healing capability. In addition, the hydrogel has good antioxidant effect which eliminates cell oxidative stress and protects cells from ROS (reactive oxygen species) damage, thereby being beneficial to accelerate wound healing after tumor surgery. The hydrogel also has strong ultraviolet absorption capacity to protect tissues from ultraviolet radiation. In addition, Fe^3+^ can induce angiogenesis, inhibit the expression of TNF-α and up-regulate the expression of CD31 to promote wound healing and tissue repairment and plays an important role in the comprehensive postoperative treatment. Besides, after the Fe^3+^ is added into hydrogel, its photothermal performance can be significantly increased, the photothermal conversion efficiency is further improved, and the ideal killing effect of residual lesions after tumor surgery is achieved through the photothermal effect. Luo et al. developed an ideal multifunctional hydrogel with abundant functions such as injectability, ultraviolet shielding, wound healing promotion, and tumor recurrence inhibition. This work has confirmed the feasibility of a multifunctional platform for comprehensive post operation treatment which may obtain good application potential in future clinical practices. Although PTT could not substitute surgery, chemotherapy, or radiotherapy to become an independent tumor treatment method, it will be a supplement for higher treatment efficiency. Therefore, the treatment of tumors by increasing the local temperature of the lesion may have great potential in treating tumor in the future.

#### Iron-based hydrogel for MHT

MHT generally refers to the heat released by a specific inductive medium during exposure to an alternating magnetic field (AMF) with appropriate frequency and amplitude [111]. The magnetic field is highly selective to the human body, so it causes little harm to health and can effectively perform high-specific hyperthermia therapy on deep and inaccessible tumors [112, 113]. In addition, hyperthermia enhances the effect of chemotherapy through surgery [114], achieving reliable tumor killing effect. Therefore, iron-based hydrogel with magnetic induction is an ideal platform for tumor postoperative treatment.

**Fig. 6:**
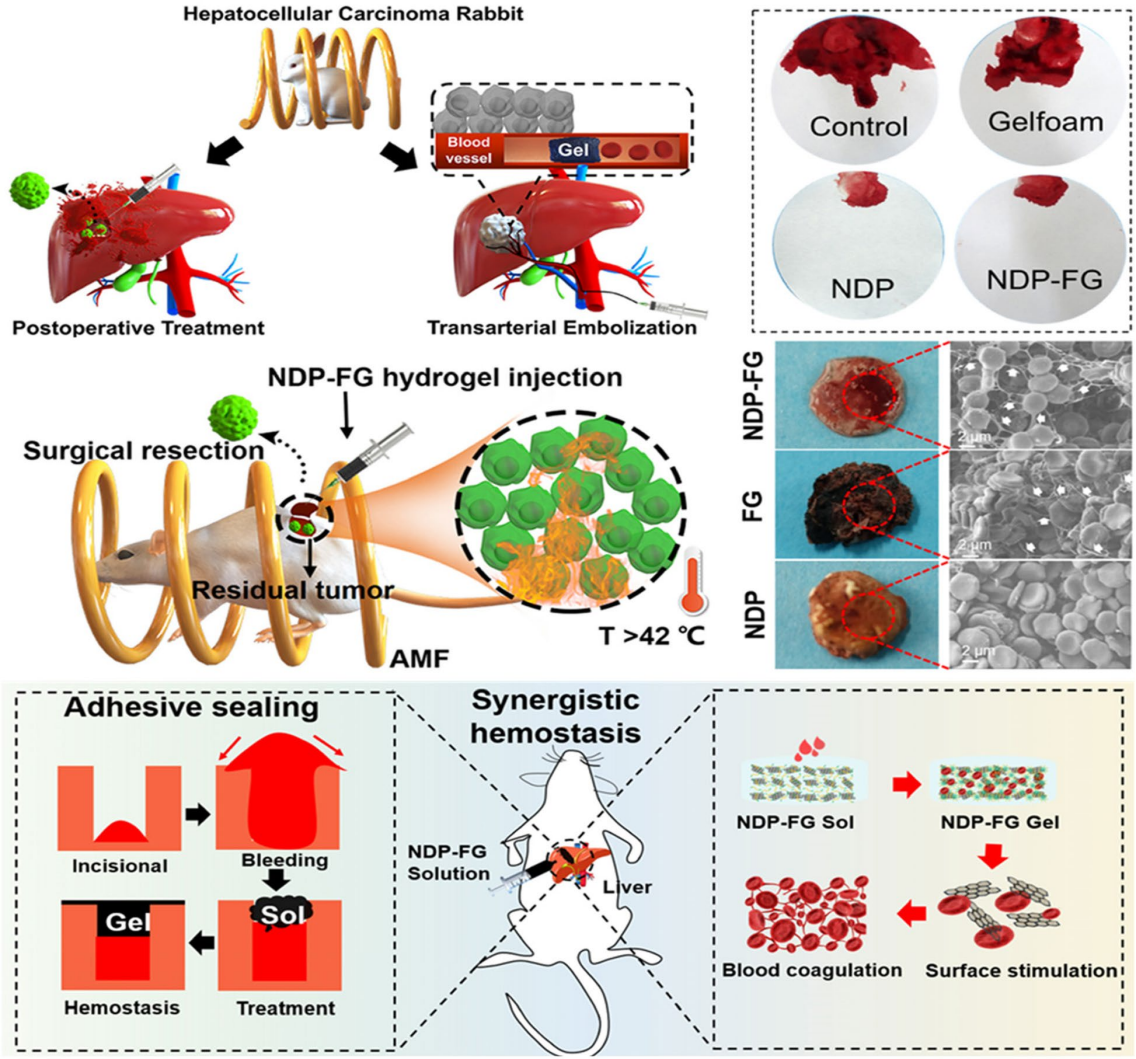
Schematic diagram shows the role of hydrogel in hemostasis, vascular embolization and MHT in postoperative treatment for HCC. Reproduced with permission from Ref. [115]

Yan et al. synthesized an in situ formed magnetic hydrogel in response to body temperature, which is composed of iron oxide nanoparticles (Fe_3_O_4_@rGO, denoted as FG), used for hepatocellular carcinoma (HCC) postoperative treatment (Fig. 6) [115]. The addition of FG improved the mechanical properties of the hydrogel. Otherwise, under the action of AMF, FG nanosheets contributes to the effective MHT after liver cancer surgery while the effect on normal cells is negligible. The hydrogel also has advantages in hemostasis and vascular embolization. When blood interacted with the FG nanosheets in the hydrogel, the negative charge on the FG enhanced the interface stimulation to red blood cells and improved the coagulation ability. Thus, in the obtained hybrid hydrogel, the dopamine group functionalized polymer matrix can seal the wound and FG contributes to improve coagulation [116, 117]. At the same time, the hydrogel is stable in injectability, so it can accurately embolize the blood vessels around the focus. The purpose of treatment is achieved by embolizing the blood vessels around the lesions, reducing blood supply and promoting the atrophy of liver cancer lesions. These combined effects effectively enhance hemostasis after liver cancer and improve the final survival rate.

**Fig. 7:**
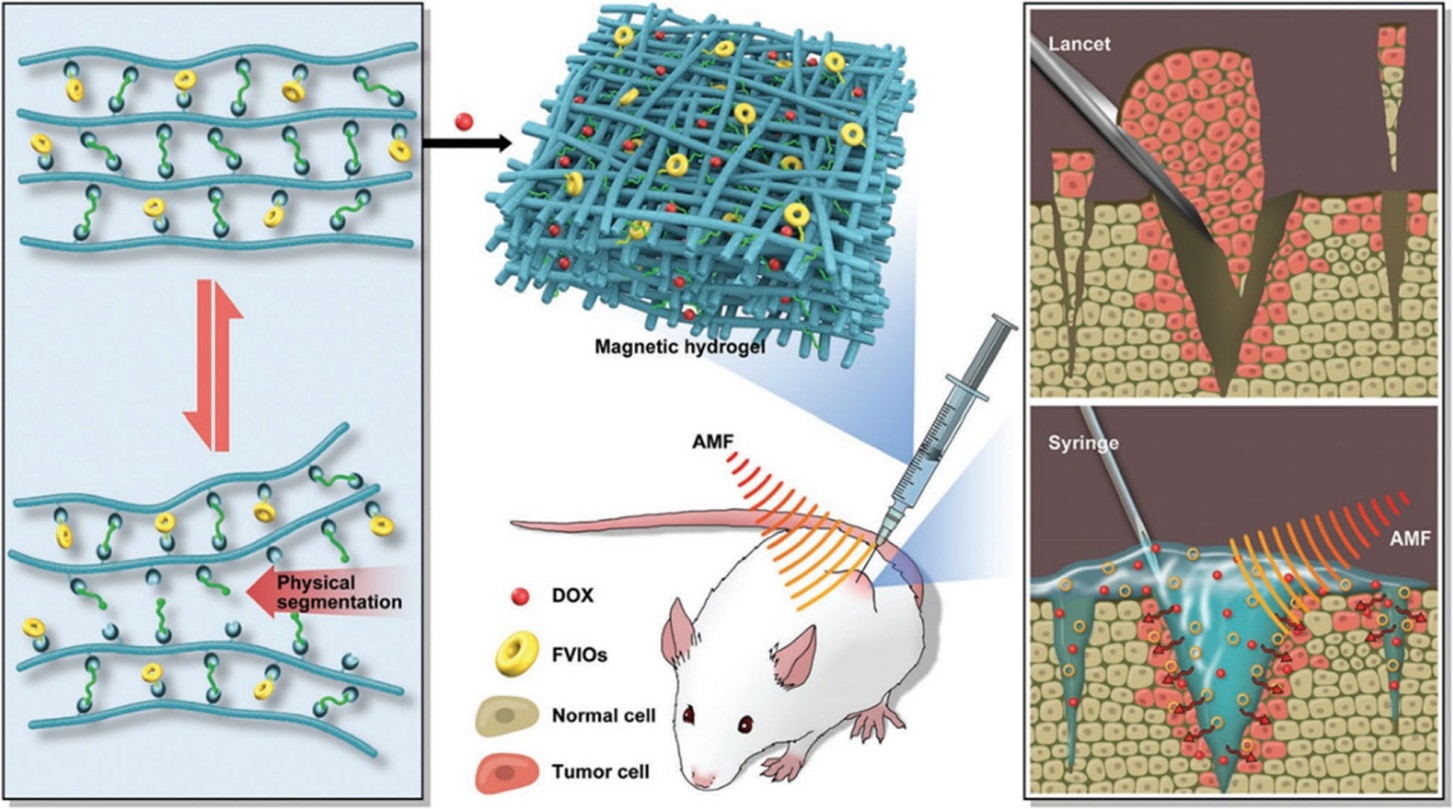
Schematic diagram illustrates FMH with optimal adaptive functions for breast cancer postoperative treatment. Reproduced with permission from Ref. [100]

In the experiment from Gao et al., a new adapting magnetic hydrogel using ferromagnetic vortex-domain iron oxide (FVIOs) incorporated onto chitosan-based dynamic hydrogels and FVIO-functionalized hydrogel (FMH) was fabricated by the grafting-onto method (Fig. 7) [100]. The doping of FVIO in the hydrogel optimized the physical properties of the magnetic hydrogel, with rapid gelling, self-healing, and self-conforming abilities, endowing the hydrogel with good injectability. FMH has good adaptability for the easy infiltration into the small gap between the residual lesions after surgery, so it can perfectly cover the tissue defects after tumor resection surgery to ensure the selective release of the drugs carried in the hydrogel and achieve good therapeutic effect. Fe_3_O_4_’s primitive cubic inverse spinel structure is the magnetic source of FIVO. Attributing to their unique magnetic reversal process from a vortex-state to onion-state, FVIO perform superior heat induction capability [118]. With AMF stimulation, FMH showed good magnetocaloric property, increasing the temperature of the surrounding environment and killing the residual tumor cells in the wound rapidly. In addition, magnetic hyperthermia generated by hydrogel could promote the delivery of carried DOX to the nuclei of cancer cells, significantly enhancing the efficacy of chemotherapy. In summary, FMH can promote effective chemo-magnetocaloric synergistic treatment and effectively prevent tumor recurrence after surgery [100]. Compared with superparamagnetic iron oxide nanoparticles (SPIOs)-functionalized magnetic hydrogel, the possible side effects of the traditional SPIO-based magnetic hydrogel has been overcome with better self-adaptability. Moreover, under the stimulation of external AMF, the hydrogel containing FIVO shows superior stability to provide a long treatment period, while showing high induction heating and significant rheological properties. In addition, only a lower concentration of FVIO is required to show the same thermal therapeutic effect as SPIO.

In summary, the doping of the Fe in the hydrogel can be used as a bridge to connect the magnetocaloric effect and the controlled release of drugs, therefore the heat can be released under an external magnetic field through the para-magnetism of the Fe element to kill the tumor. Furthermore, the controlled release of drugs in chemotherapy can be promoted through the raise of temperature, so that the comprehensive treatment after the tumor surgery can be achieved.

#### Iron-based hydrogel for CDT

CDT is a new ROS-mediated cancer treatment method based on in situ Fenton reaction in tumor site [102, 119, 120]. In the CDT process, metal ions such as Cu [121], Fe [122], Mn [123], catalyzed excessive endogenous H_2_O_2_ in tumor microenvironment (TME) by Fenton or Fenton-like reaction to generate highly toxic •OH [124], which can specifically cause the death of tumor cells.

**Fig. 8:**
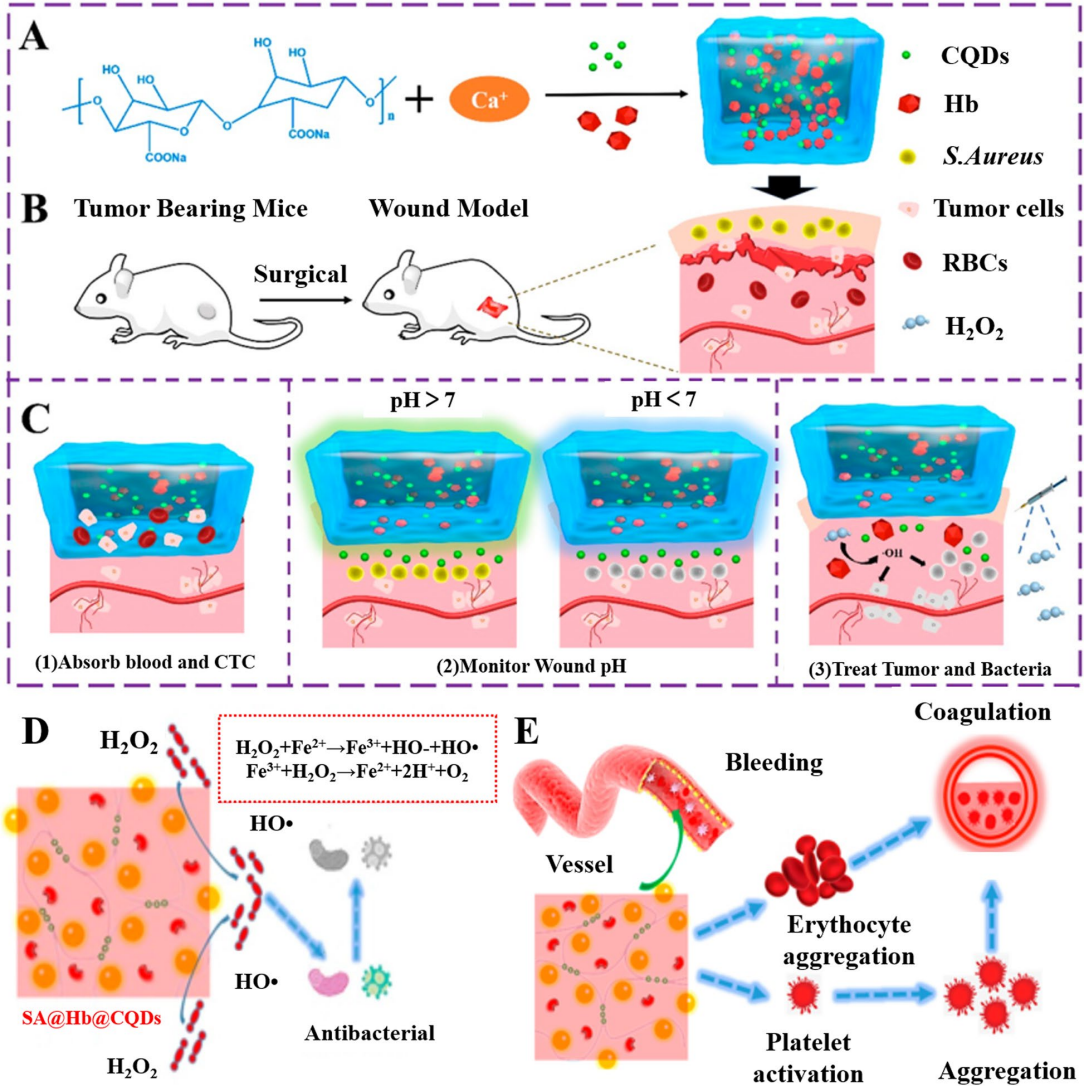
Schematics illustrate the potential application of the inhibiting tumor recurrence and infection. Reproduced with permission from Ref. [125]

In the experiment from Zhang et al., a multifunctional sodium alginate (SA) hydrogel immobilizing hemoglobin (Hb) and pH-sensitive fluorescent changing carbon quantum dots (CQDs) was designed [125] (Fig. 8). This is a new multifunctional implant with the effects of preventing tumor recurrence and infection after tumor resection, detecting pH of tumor microenvironment (TME) and stopping bleeding. With the tumor endogenous H_2_O_2_, the Fe^2+^ in Hb was able to generate toxic •OH by Fenton reaction, effectively killing the residual recurrent cancer cells and the infected bacteria which makes the hydrogel a good method for comprehensive postoperative treatment. First, the hydrogel has good water absorption ability to absorb exuded blood, thereby improving the local concentration of hemostatic factors, promoting thrombosis, reducing blood loss, and playing a role in effective hemostasis after surgery. Secondly, hydrogel can quickly capture the disseminated tumor cells into micropores, reduce the circulating tumor cell (CTC) level and the risk of tumor recurrence after operation. Thirdly, immobilized hemoglobin is a natural protein in human body, which is composed of four heme groups. Due to Fe^2+^ in the center of heme groups, Hb can undergo Fenton reaction in TME where H_2_O_2_ is over-expressed, producing •OH with high cancer cells toxicity and broad-spectrum antibacterial activity to reduce the risk of recurrence, metastasis, and wound infection in the short term after radical tumor resection.

#### Iron-based hydrogel for synergistic CDT and PTT

CDT and PTT have a broad prospect in enhancing the anticancer effect. However, monotherapy equipped with a single model of anticancer efficacy is generally insufficient to induce an adequate therapeutic response. When PTT is combined with CDT, a synergistic therapeutic effect will be generated by PTT which can not only kill cancer cells, but also accelerate the generation of •OH in CDT [126]. The toxicity and side effects of drugs can be reduced at the same time, thereby achieving the ideal clinical effect [127].

**Fig. 9:**
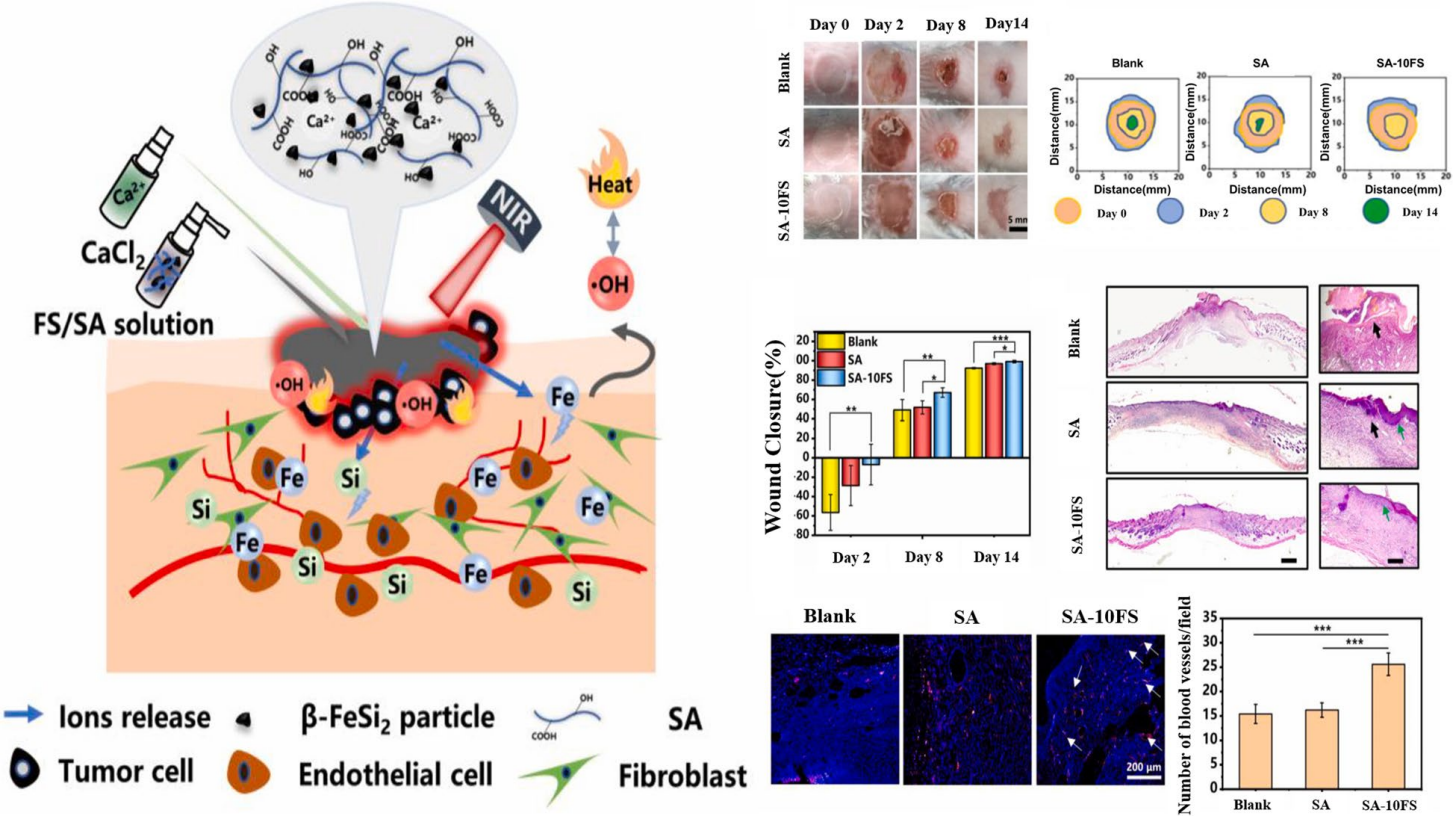
The schematic diagram of the preparation and function of sprayable FS/SA hydrogel. Reproduced with permission from Ref. [89]

Ma et al., fabricated a sprayable FS/SA composite hydrogel which was composited by β-FeSi_2_ (FS) and CaCl_2_. In this study, FS was utilized as a bioactive material for tumor postoperative recurrence inhabitation and skin wound healing for the first time (Fig. 9). Due to the local surface plasmon resonance phenomenon of particles, FS has a good photothermal effect with about 28.9% photothermal conversion efficiency that proportional to the content of Fe in the hydrogel, resulting in strong visible and near-infrared light absorption. FS can absorb the photon energy at 808 nm, triggering an electronic transition. The excited electrons can combine with the holes in a nonradiative manner to generate phonons to release the thermal radiation to the surrounding environment. In addition, the release of Fe ions in FS could trigger the Fenton reaction to produce •OH in the weak acidic environment, killing residual tumor cells and antibacterial. Therefore, due to the presence of FS, the prepared FS/SA hydrogel has excellent photothermal and chemical kinetic properties which can be flexibly controlled by adjusting the content of FS and the laser power density. In addition, the release of Fe ion in the hydrogel was also involved in the promotion of angiogenesis and wound healing. After being released from the hydrogel, Fe^2+^ is easily oxidized to Fe^3+^, the increased proportion of Fe^3+^ inhibits the expression activity of prolyl hydroxylase. The degradation of hypoxia inducible factor-1 (HIF-1α) proteasome increases the level of HIF-1α. The stable expression of HIF-1α can significantly up-regulate the expression of VEGF, thereby inducing angiogenesis. Fe ions can promote the migration and differentiation of endothelial cells by promoting the high expression of eNOS. In conclusion, FS has excellent biological activity in promoting endothelial cells migration, differentiation, as well as angiogenesis in vitro [89].

**Fig. 10:**
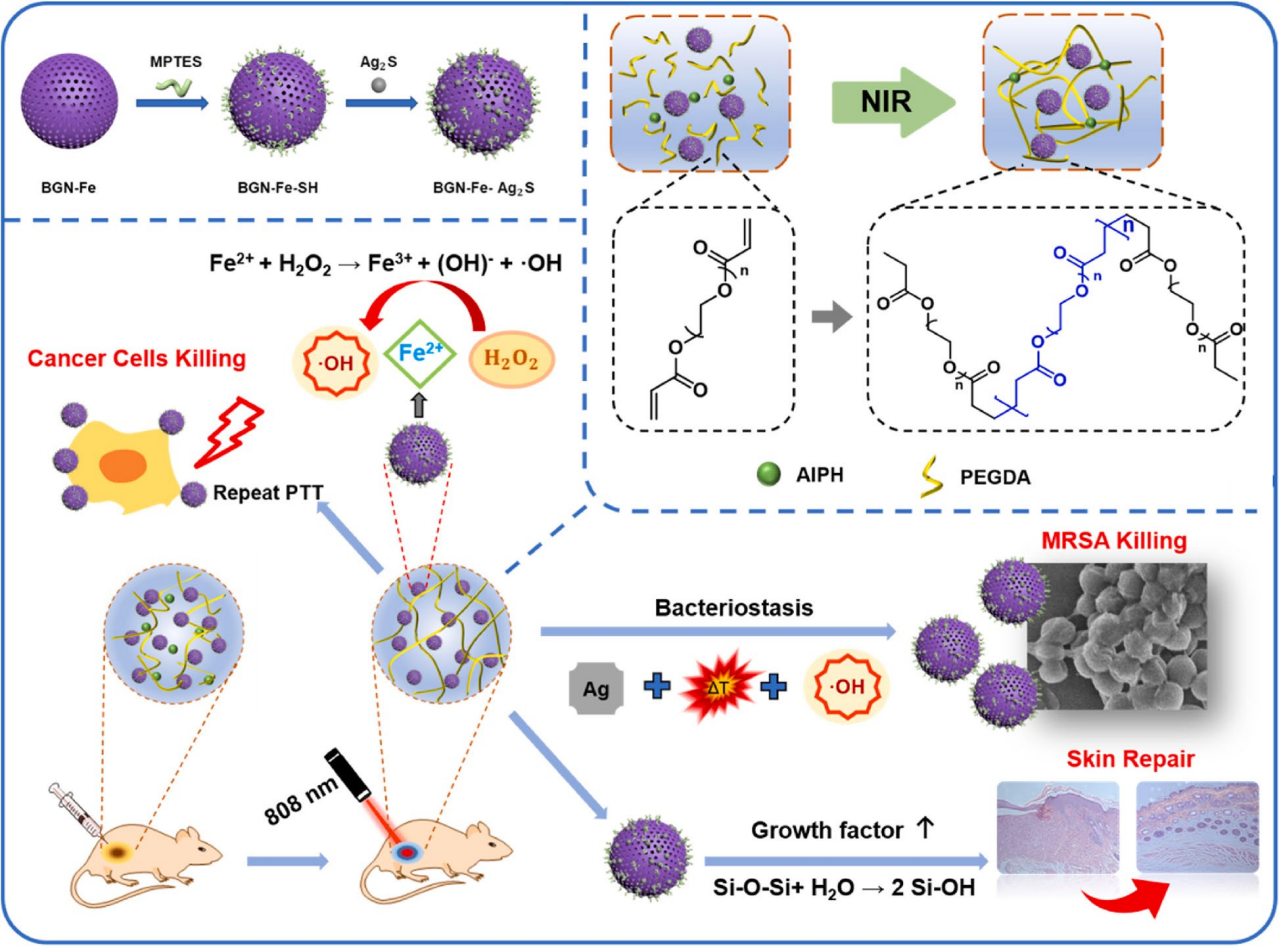
Schematic illustration of the preparation of BGN-Fe-Ag_2_S hydrogels and their application in anticancer, antibacterial, and skin repairment. Reproduced with permission from Ref. [99]

Huang et al. synthesized a bioactive nanocomposite hydrogel by incorporating Ag_2_S nanodots conjugated Fe-doped bioactive glass nanoparticles (BGN-Fe-Ag_2_S) into biodegradable PEGDA and AIPH solution (Fig. 10) [99]. Under the irradiation of laser, Ag_2_S can release a certain amount of hyperthermia, induce the decomposition of AIPH, release alkyl free radicals, and then initiate the polymerization of PEGDA which leads to the gelation of hydrogel in the lesion and fixes BGN-Fe-Ag_2_S in the lesion. Based on the over-expressed H_2_O_2_ in the inflammatory microenvironment, •OH are generated by that Fenton reaction, and BGN-Fe was used as a growth promoter of wound tissues together with CDT agent to inhibit tumor growth and bacterial proliferation. Besides, nanocomposite hydrogel can be hydrolyzed under laser irradiation to produce growth factors and facilitate wound healing by promoting granulation tissue growth and collagen deposition. As an ideal growth promoter and chemokinetic therapeutic agent, iron-based hydrogel can realize the functional controllability of anti-infection, residual lesions eradication after tumor resection, enhanced wound healing and scar repairment. In general, injectable iron-based hydrogels are one of the promising candidates for mitigating postoperative side effects and improving patients’ quality of life.

### Application of Iron-based hydrogels in other biomedical aspects

Iron-based hydrogels have great development potential in the field of tumor treatment which occupy an important position in the chemotherapy, MHT, PTT, CDT, chemokinetic therapy, and other fields. Moreover, because of the good water absorption, tissue compatibility, low cytotoxicity, together with the effects in promoting angiogenesis and inhibiting bacteria, iron-based hydrogel also plays an important role in tissue regeneration [128, 129], chronic wound healing promoting [130–132], anti-infection [133, 134], controlled drug release [135–137], image diagnosis [138]. Table 2 have listed the recent studies on the various clinical biological applications of iron-based hydrogels.

Fe (III)@TA microsphere, formed by coordination of ferric ions and tannic acid complexes, is one of the simplest metal-polyphenol networks [139]. Fe (III)@TA coating is a good platform to trigger the endosomal escape of nanoparticles, thus enhancing the treatment efficiency of bioactive factor [140]. Meanwhile, the drug loading efficiency and the durability of the peptide can be improved without damaging the peptide of normal structure, [141], making it commonly be used for drug delivery. In the experiment of Chen et al., a gelatin methacrylate (GelMA)/oxidized hyaluronic acid (OHA)/galactosylated chitosan (Gal-CS)/Fe (III)@TA@IGF-2 200 (TA200) hydrogel loaded with insulin-like growth factor 2 (IGF-2) was manufactured for liver regeneration [92]. Fe (III)@TA microspheres with uniform particle size are used as IGF-2 absorber to realize a long-term stable release, GelMA/OHA/Gal-CS@TA200 can meet the demands of liver tissue damage repairment through good biocompatibility and sustained release characteristics. In a conclusion, iron-based hydrogel may have the development potential in the fields of promoting liver regeneration and artificial liver in the future.

**Table 2:** Iron-based hydrogels for other biological application

Host material	Existence	Function	Indication	Treatment strategy	Ref.
Tunicate-inspired gelatin-based hydrogel	Fe^3+^	tuning gelation time; rheological property and self-healing ability by adjusting the composition	diabetes wounds	wound healing in a diabetes	[88]
Oxidized sodium hyaluronic acid (HA-CHO) and hydrazided hyaluronic acid (HHA))	Fe^3+^	enhancing the cohesion and interface adhesion; providing mechanical support and promoting angiogenesis	myocardial infarction	treating myocardial infarction	[90]
Poly (vinyl alcohol), nano-hydroxyapatite)	Fe_2_O_3_	promoting the proliferation and differentiation of bone mesenchymal stem cells; stimulating chondrocyte-related gene expression	cartilage tissue engineering	cartilage regeneration	[91]
Chitosan/PEG hydrogel	Fe_3_O_4_	increasing the temperature under the AMF	bone regeneration	MHT	[93]
Gelatin methacrylate /oxidized hyaluronic acid /galactosylated chitosan	Fe^3+^	controlling the release of IGF-2; promoting hepatocytes regeneration	hepatocytes repairing	Liver regeneration; artificial livers for drug screening	[92]
Vancomycin-agarose-ferric tannate hydrogel	Fe^3+^	generating local hyperthermia; promoting the spatiotemporal release of antibiotics	wound disinfection	PTT	[94]
Gelatin/Fe_3_O_4_/celecoxib	Fe_3_O_4_	accelerating the release of celecoxib; increasing the temperature	tendon tissue injury	MHT-Chemotherapy	[95]

In the field of cartilage tissue repairment, iron-based hydrogels also have unique advantages. The hydrogels have 3D structure with high hydrophilicity and good biological which is similar to cartilage tissue, making it easier for stem cells to embed and recruit enough endogenous cells (especially stem cells) to induce cartilage regeneration. The bioactive substances can be added into the hydrogel base to simulate extracellular matrix (ECM) which promotes cell adhesion, proliferation, and differentiation. Wang et al. manufactured poly (γ-glutamic acid) (γ-PGA) hydrogel and Fe^3+^ ligand coordination to repair cartilage defect [129]. The secondary crosslinking of Fe^3+^ with the carboxyl of the γ-PGA further improves the crosslinking density of the hydrogel and increases the mechanical strength of the hydrogel [142], promoting proliferation and directed differentiation of mesenchymal stem cells (BMSCs) [143–145], inducing cartilage specific gene expression to enhance damage repair and cartilage tissues regeneration. Besides, The Fe^3+^ is involved in the induction of BMSCs differentiation into cartilage tissue which provides a novel idea for BMSCs cartilage formation and clinical cartilage regeneration.

Iron-based hydrogel is also an ideal biological dressing for preventing infection, promoting wound closure and tissue repairment due to its excellent biocompatibility and adjustable structure [146]. For example, in the EDTA-Fe^3+^ complexes crosslinked hyaluronic acid designed by Tian et al. [147], hyaluronic acid was degraded by hyaluronidase secreted from bacteria to release Fe^3+^ complex around the bacteria which was quickly absorbed by the surrounding bacteria and reduced to Fe^2+^. Then Fe^2+^ reacted with H_2_O_2_ to generate •OH, destroying proteins and nucleus to realize effective anti-infection [148, 149]. In addition, Fe^3+^ was used as a part of the physical cross-linking agent to fill the whole hydrogel, thereby greatly increasing the loading of the antibacterial agent and prolonging the effective period, enabling the hydrogel to continuously release the antibacterial agents until the hydrogel are completely decomposed or the bacteria surrounded are completely killed without inducing the drug resistance of the bacteria [150, 151].

Compared with the metal-free hydrogel (Table 3), the addition of Fe element improves and the clinical application potential of the hydrogel. For example, the intrinsic physical properties of the hydrogel substrate, such as the strength, injectability, self-healing property, can be greatly improved. In addition, the sensitivity to external stimulation, the drug carrying capacity, and the controlled release ability of the hydrogel are strengthened. To enrich the functions of the hydrogel, such as the photothermal effect, the magnetocaloric effect, and the integration of multiple functions, Fe can be added into the hydrogel in different forms. Compared with other metals (Table 4), Fe is an important trace element that can be digested and absorbed by the human body after the treatment. Meanwhile, the threshold of Fe poisoning is much higher than other metals, thus, obvious side effects will not be introduced and the metabolic burden of Fe to the human body is much smaller as well. In addition, Fe can induce Fenton reaction and produce amount of ROS to improve hydrogel’s antibacterial property and tumor killing function with the reduced risk of carcinogenicity than other metals. Fe is involved in the synthesis of a variety of active ingredients in the human body, and these bioactive ingredients can also be directly combined with the hydrogel to significantly improve the overall biocompatibility of the hydrogel. Meanwhile, Fe’ s magnetic properties can play an important role in the fields of Magnetic Resonance Imaging (MRI). At the same time, Fe has the unique characteristics of promoting angiogenesis and blade healing, especially in the fields of anti-infection of major surgical wounds and promoting healing. Therefore, the development of iron-based hydrogels in the clinical application should be a hot topic in future medical research.

**Table 3:** Metal-free hydrogels for postoperative cancer therapy

Material	Function	Indication	Treatment strategy	Ref.
Methylcellulose hydrogel	preventing post-surgical breast cancer recurrence; photothermal performance	breast cancer	PTT; breast reconstruction	[67]
Penetrating peptide (CRGDK)-modified doxorubicin-based prodrug nanoparticles	realizing tumor-specific targeting; increasing tunable loading capacity; controlled drug releasing;	breast cancer	Local chemotherapy;	[152]
Raltitrexed hydrogel	inhibiting thymidylate synthase with remarkable selectivity;	post-surgical cancer	Chemotherapy	[153]
Hyaluronic acid-based hydrogel	realizing simultaneous delivery and sustain release of PTX and EPB for preventing postoperative recurrence and metastasis of breast tumors	breast cancer	Chemotherapy	[154]
Poly(ethyleneglycol)–poly(ε-caprolactone)–poly(ethylene glycol)	enhancing anti-tumor efficacy in the local region; decreasing systemic toxicity, and improve the patient compliance	breast cancer	Chemotherapy	[155]
Bis(2-methacryloyl)-oxyethyl disulfide and [2-(Methacryloyloxy)-ethyl]dimethyl-(3-sulfo-propyl)-ammonium hydroxide	down-regulating the expression of anti-apoptosis genes and up-regulating the expression of apoptosis genes.	osteosarcoma	temporary filler; PDT- Immunotherapy	[156]
Chondroitin sulfate multi-aldehyde, branched poly-ethylenimine and BPEI conjugated graphene	realizing chemo-photothermal therapy; controlled drug delivery	breast cancer	PTT-Chemotherapy	[157]
Aldehyde hyaluronic acid and the carboxymethyl chitosan	controlled drug delivery	breast cancer	Chemotherapy	[6]
Temozolomide + O^6^-benzylamine hydrogel	inhibiting the recurrence of TMZ-resistant glioma; responding to MMPs enzyme; releasing TMZ and BG; enhancing the efficiency of TMZ to inhibit glioma growth	gliomas	Chemotherapy	[61]
Personalized tumor lysate derived hydrogel	Stimulating the antitumor immune response for the inhibition of residual tumor cells;	pancreatic cancer	Immunotherapy	[158]

**Table 4:** Other metal-based hydrogels for postoperative cancer therapy

Metal	Function	Indication	Treatment strategy	Ref.
Cu	generating ROS, killing the residual cancer cells, preventing the orthotopic tumor recurrence, and realizing local antisepsis	lung adenocarcinoma	CDT-PPT-Immunotherapy	[159]
Mn	alleviation of tumor hypoxia; photothermal hyperthermia; anti-infection	cutaneous cancer	CDT; PPT	[160]
Mn	Decomposing H_2_O_2_ to produce O_2_; PTT; loading GOx	skin tumors	PTT-Starvation therapy;	[161]
Ge	Increasing drug-loading capacity and multi-responsive; increasing good biocompatibility, and drug-release behavior;realizing multimodal imaging-guided treatment	Breast Cancer	PPT-Chemotherapy	[162]
Ca	enhancing the mechanical properties of the patches; reducing their swelling ratio	pancreatic cancer	Chemotherapy	[163]
Ti	acting as photosensitizer	skin tumors	PTT-PDT	[164]

## Conclusion, discussion, and future perspective

Postoperative cancer therapy still faces great challenges due to the difficulties in tumor recurrence and metastasis inhibition. As personalized therapy have become an important goal of tumor research, hydrogels have shown great inhibition ability in cancer therapy. Due to Fe element’s unique functions and good biocompatibility, iron-based hydrogels displayed huge potential in postoperative cancer therapy. This review has summarized various synthesis methods and construction principles of iron-based hydrogels and highlighted the latest progress of iron-based hydrogels in postoperative tumor therapy, including chemotherapy, PTT, PDT, CDT, MHT, and multimodal combination therapy. Although some progresses have been achieved so far, there is still a long way to go until wide iron-based hydrogel applications are available in clinic and some challenges is still waiting to be fully addressed.

As it is known that iron-based hydrogels have advantageous properties in clinical transformation. For example, due to the simple preparation and readily available raw materials, the production cost of iron-based hydrogel is relatively low to reduce the economic burden on patients. As a good drug carrier, hydrogel can be loaded by a variety of bioactive drugs, such as growth factor, anti-inflammatory factor, or chemotherapy drugs, and integrate various of functions to achieve comprehensive treatment after radical surgical treatment of tumors. By virtue of the morphological changes of the hydrogel under certain stimulations, the tiny defects after tumor surgical resection can be fully covered to reduce the risks of tumor recurrence and metastasis. With the excellent water absorption capacity, hydrogel can be used to absorb tissue exudate around the wound when it is covered on the operation site to inhibit or relieve pain, control inflammation, and reduce scar formation. Hydrogel can accurately cover defects of surgical and isolate the tissue from the external environment which can be used to effectively reduce infection. With the good plasticity, hydrogel will not generate obvious stress and side effects to the surrounding normal tissues when it is applied to local areas.

Iron-based hydrogels have shown great advantages in the field of cancer nanomedicine, however, further clarifications should be made before clinical application. For example, at present, in the preparation of bioactive hydrogel, bioactive components have strict transportation and storage requirements which may be inactivated at certain ambient temperature and will affect the synthetizing process and character of hydrogel. However, there are few studies about the preservation conditions and shelf life of bioactive hydrogels. In addition, it is difficult to keep the hydrogels at suitable storage conditions when that are used by patients themselves, affecting the curative effect of hydrogel and reducing the patients’ treatment compliance. Intravenously injected nanodrugs may have relatively easy access to the intact tumor microenvironment and be sufficiently stimulated by the tumor microenvironment. While the postoperative inflammatory environment may be detrimental to the stimuli-responsive drug release of iron-based hydrogels which previously only occur in the tumor microenvironment. From this perspective, it is necessary to re-examine the hydrogel construction strategy.

As the post-surgery residual may disperse at multiple sites, the single beam’s NIR light of PTT/PDT of some hydrogels cannot reach tumor location. Therefore, the ideal photo-responsive therapeutic effect is hardly achieved. Due to the short application window of hydrogel and easy absorption by non-tumor tissues, it may cause certain immunogenicity. Furthermore, some components of hydrogel have the similar functions as the initiators of partial positive and negative feedback reactions in human body fluids, potentially causing lesions such as inflammatory reactions [165] and abnormal coagulation, even become life-threatening in severe cases. Its biosafety, biodegradability, metabolic rate, and cytotoxicity should be further studied. At the current stage, the short-term biocompatibility of hydrogels has only been evaluated in animal models without the long-term safety of hydrogels. The pharmacokinetics are easily elucidated in vitro, whereas the release rate and metabolism in vivo remain unclear, and the drug release per unit time is difficult to be precisely controlled. At the same time, there may be liver-kidney metabolism and excretion pathways of hydrogel, and kidney excretion pathways mostly depend on the diameter of the drug. Hydrogels are mostly macromolecular cross-linked structured with relatively large molecular weight and molecular size which may affect their metabolism and excretion efficiency [166]. In addition, hydrogels are composed by multiple components, the further combination with multiple therapeutic strategies makes the system be complicated and hard for scale-up and quality control. Some injectable hydrogels have converted into gel prematurely in the needle, thus, controlling the activation condition of hydrogels at more precise temperatures to reduce the risk of premature gelation.

Although the incorporation of Fe into the hydrogel can enrich the function of the hydrogel, its effect is mostly proportional to the content. When Fe exceeds a certain dose, iron poisoning might be caused. Furthermore, the design to involve the target of iron-based hydrogels is also relatively difficult because postoperative treatment is generally injected locally. Unlike the ordinary nanomedicines that can be targeted and accumulated inside the tumor after intravenous injection, the hydrogels are applied after surgery. After the site gels, some of the drug components are absorbed by the tumor rather than the hydrogel. In the future, the development of iron-based hydrogels with high stimuli responsiveness, high biocompatibility, high biodegradability, and high targeting should be focused. Although iron-based hydrogels have made great progress in the postoperative treatment of tumors, from the perspective of clinical transformation of tumor treatment drugs, hydrogels cannot completely replace the existing postoperative adjuvant therapy. Therefore, many factors restrict the clinical application of hydrogels which are also the challenges faced by many new polymer materials in the application.

In practical clinical application, the comprehensive treatment plan for postoperative patients should be determined according to their pathological type, but pathological results cost a long time. Meanwhile, the usage of some chemotherapeutic drugs such as anti-angiogenic drugs need several months interval from surgery which will lead to the inability to achieve the timely application of hydrogel to the surgical site during the operation. Second, along with the peristalsis of the gastrointestinal tract, when the hydrogel is applied to the pelvic and abdominal cavities, the existing site of the hydrogel may be changed, resulting in the decrease of the drug concentration at the surgical site of the tumor and affecting the therapeutic effect. Hydrogel has a certain viscosity, and as a foreign body, it will aggravate the adhesion after pelvic and abdominal surgery and increase the risk and difficulty of reoperation. In the recovery period of surgery, the presence of hydrogel may affect the recovery of gastrointestinal peristalsis, causing gastrointestinal flatulence, abdominal pain, and other discomforts, and even lead to serious postoperative complications such as intestinal obstruction.

Most of the experiments focused on the characterization and efficacy of hydrogels, but the studies in practical clinical application are still rare. And most of them are external dosage forms for anti-infection and promoting tissue healing. Hydrogels that are applied in vivo or used for tumor treatment are rare. As mentioned above, there are still many practical difficulties in clinical application that restrict the potential application of hydrogels and further limit the development of clinical human experiments. At present, the research on hydrogels mainly focuses on the level of cell and animal experiments, and the number of animal experiments is relatively small with a lack of safety evaluation and it does not meet the standards for human experiments. In addition, most of the hydrogel drugs that have entered clinical practice or clinical trials are external reagents, and very few hydrogels are given in vivo with short observation period. Therefore, there are inadequate clinical trial-based medical evidence on the long-term adverse reactions of its application. As a new type of biomaterial, most doctors and patients still have skepticism and wait-and-see attitude for the use of hydrogel in vivo which will cause certain difficulties in the recruitment of observation subjects for clinical trials. Finally, to ensure the reliability of hydrogel clinical experiments, it will cause practical problems such as long observation period, harsh entry conditions, and difficulty in tracking.

The hydrogels still have great potential in the future clinical practice. Firstly, the selected drugs for chemotherapy after tumor surgery are related to the pathological type of tumor. The existing research mostly focuses on the combination of a hydrogel substrate and one or more fixed chemotherapy drugs. To solve the arbitrary collocation of the hydrogel substrate and the chemotherapy drugs as well as the artificial and accurate formulation of the drug content may be the trend and focus of hydrogel development in the future. Secondly, most of the existing experiments focus on solid tumors such as breast cancer, lung cancer and skin cancer, while few studies are about non-solid tumors such as leukemia which means that the hydrogel has great development potential and prospects in the field of non-solid tumor treatment. With the unique biocompatibility of hydrogel, it may be a new idea of treatment. Hydrogels are same as human tissue matrix which have natural advantages in carrying bioactive substances, enzymes, DNA and RNA, and have great potential in the fields of developing tumor vaccines, immunotherapy, and gene therapy. In addition, tumors grow at sites protective barrier structures are difficult to be treated by conventional administration routes, the utilization of hydrogel may be a novel method. Future research can also focus on achieving high aggregation in the tumor microenvironment by virtue of the pH responsiveness, photothermal effect and other characteristics, reducing the hepatorenal toxicity and irritation of the existing contrast agents, and realizing the application in accurate cancer detection. The injectable multidisciplinary hydrogel-based delivery systems for the co-delivery and sequential release of different therapeutic agents are expected to maximize the overall therapeutic efficiency of cancer therapies and accelerate their clinical translation.

In the future, in terms of the composition of hydrogels, it is necessary to explore a simpler method with simpler components but multiple functions, for example, the development of iron-based hydrogels with high stimuli responsiveness, high biocompatibility, high biodegradability, and high targeting should be focused. In treatment methods aspect, besides treating the tumors directly with iron-based hydrogels, alleviating the inflammatory state in the surgical site to improve the tumor microenvironment should also be a promising postoperative cancer therapy method. Because of the improvement of inflammatory microenvironment, Epithelial-Mesenchymal Transition of tumor cells can be well inhibited which can also reduce the risk of CTC to colonize in distant organs. In addition, there is a substantial requirement to design appropriate hydrogels with the combined therapy strategies, including chemotherapy, immunotherapy, PTT, PDT, gene therapy, radiation therapy, and so on, improving postoperative tumor therapy in various synergistic modalities.

In summary, although iron-based hydrogels have made great progress in the postoperative treatment of tumors, from the perspective of clinical transformation of tumor treatment drugs, iron-based hydrogels still exist many limitations. It should not be ignored that these limitations prevent their further practical application. Fortunately, things are looking up, hydrogel is developing very rapidly under the current situation with more and more research force. If the shortcomings found in the existing research can be optimized, the functions can be centralized, and the advantages can be reasonably exerted, iron-based hydrogel will play an important role in future cancer therapy.

## Data Availability

Not applicable.

## Abbreviations

NIR: Near infrared light
·OH: Hydroxyl radical
PTT: Photothermal therapy
MHT: Magnetic hyperthermia therapy
PDT: Photodynamic therapy
CDT: Chemo-dynamic therapy
CAT-CHIT: Chitosan-catechol based hydrogel
DOX: Doxorubicin
DTX: Docetaxel
MSH: Magnetic supramolecular hydrogel
PTX: Molecule taxus chinensis
ACMF: Alternating current magnetic field
VEGF: Vascular endothelial growth factor
GPDF: Citrate-iron hydrogel scaffold
PCD: Poly (citric acid-ethylene glycol) (PCG)-dopamine
GelMA: Gelatin/methacrylic anhydride
ROS: Reactive oxygen species
TNF-α: Tumor necrosis factor-α
AMF: Alternating magnetic field
HCC: Hepatocellular carcinoma
FG: Iron oxide nanoparticles
FVIOs: Ferromagnetic vortex-domain iron oxide
FMH: FVIO-functionalized hydrogel
SPIOs: Superparamagnetic iron oxide nanoparticles
TME: Tumor microenvironment
SA: Sodium alginate
Hb: Hemoglobin
CODs: Carbon quantum dots
CTC: Circulating tumor cell
FS: β-FeSi_2_
HIF-1: Hypoxia inducible factor-1
eNOS: Endothelial Nitric Oxide Synthases
BGN-Fe-Ag_2_S: Ag_2_S nanodots conjugated Fe-doped bioactive glass nanoparticles
PEGDA: Polyethylene glycol diacrylate
OHA: Oxidized hyaluronic acid
Gal-CS: Galactosylated chitosan
TA200: Fe(III)@TA@IGF-2 200
IGF-2: Insulin-like growth factor 2
ECM: Simulate extracellular matrix
γ-PGA: Poly (γ-glutamic acid)
BMSCs: Mesenchymal stem cells
MRI: Magnetic Resonance Imaging
